# Epidemic of Non-Hodgkin Lymphoma in New Zealand Remains Unexplained

**DOI:** 10.1155/2014/315378

**Published:** 2014-04-01

**Authors:** Brian Cox, Chih-Wei Liu, Mary J. Sneyd, Claire M. Cameron

**Affiliations:** Hugh Adam Cancer Epidemiology Unit, Department of Preventive and Social Medicine, Dunedin School of Medicine, University of Otago, P.O. Box 56, Dunedin 9054, New Zealand

## Abstract

*Background*. Non-Hodgkin lymphoma (NHL) incidence rates have increased considerably in New Zealand. *Methods*. Incidence and mortality rates for NHL from 1981 to 2010 were calculated. Trends in age-specific rates were analysed and age-period-cohort models fitted to explore generation-specific changes in incidence and mortality. *Results*. NHL incidence increased by 67% for men and 74% for women between the 1981–1985 and 2006–2010 time periods in New Zealand. For women born about 1936 and men born about 1946, NHL incidence and mortality have diverged suggesting an improved prognosis for recent generations. *Conclusion*. The strong generation effects suggest that an exposure before 25 years of age is of major importance in determining the lifetime risk of NHL in New Zealand. NHL incidence rates in New Zealand will continue to increase in the future and probably more in females than males, as generations with increased risk age. Current hypotheses for the cause of NHL do not explain the trends observed. A decline in the prevalence of a protective factor may have also contributed to these trends. Examination of trends for subtypes of NHL and innovative testable hypotheses that may explain these trends are needed.

## 1. Introduction

The epidemic of non-Hodgkin lymphoma (NHL) in many countries has received considerable attention from the United States National Cancer Institute [1] and other agencies, but the reasons for the epidemic and means of its control have not been found [2]. In New Zealand, the incidence of NHL has also increased rapidly over the past 40 years and has been projected to increase further [3].

The classification of lymphoid neoplasms has changed several times since the 1940s, as appreciation of the many different forms of lymphoma has improved [4–6]. We have chosen to specifically assess trends in NHL in New Zealand. Although the changes to the classification of lymphoma have had a minor effect on the incidence of NHL overall [6], they have produced significant changes in incidence for some subtypes of NHL.

The effects of risk factors are typically illustrated by differences in risk between generations, specified by their year of birth, with exposures at young ages predominantly determining the lifetime risk of the disease. Therefore, three important and linearly dependent aspects of time require assessment: a person's age, which usually represents the duration and cumulative exposure to a causative agent, the time period of diagnosis or death from NHL, in which changes in diagnostic practice and classification, or, for mortality, changes in treatment across most age groups, can be seen, and the year of birth, by which changes in lifetime risk from different lifestyle exposures for each generation are shown. Any assessment of two or fewer of these three variables can be confounded by the third variable: interpretation is the easiest when one of the three time variables is known to be constant. However, the risk of cancer almost always increases considerably with age, so the effects of age need to be included in most assessments of cancer risk. Age-adjusted rates for different time periods largely represent changes in classification, diagnosis, or completeness of registration and that method of analysis is an incomplete assessment of the contribution of changing risk factors to the burden of cancer.

Classic graphical methods for analysing trends in cancer involve assessing, at most, two of these important contributors to changes in incidence and mortality [7]. For example, graphical cohort analysis involves plotting age-specific rates for generations identified by their year of birth and is most informative when the effects of the time period on the diagnosis or death are known to be unchanged over the time period studied. Age-period-cohort (APC) models are a statistical approach to try and separate the three etiologically important, but interdependent, time variables to provide further insights into the underlying forces influencing the burden of disease. These methods have been successfully employed over many years [8]. The linear dependence of age, time period of diagnosis or death, and year of birth creates the identification problem in statistical APC models but this is equally present, although less easily seen, in the accepted classic graphical methods of cohort analysis [7].

The changes in the classification of NHL and the 1994 transition from clinical and hospital information for cancer registration to statutory notification from pathology reports in New Zealand can be expected to have produced changes in NHL incidence and mortality. Therefore, the interpretation of changes in the risk of NHL for different generations requires the use of APC models to separate these three effects and to adjust generation effects for confounding from both age and time period.

## 2. Materials and Methods

A nationwide cancer registry has been in operation in New Zealand since 1948. The annual age-specific numbers of deaths and registrations attributed to NHL (ICD-10 rubrics, C82–C85) during the years 1981 to 2010 in New Zealand, together with estimates of the relevant population counts, were obtained from publications of the Ministry of Health [9, 10]. This time period was chosen to ensure consistent classification of NHL for the time period studied, because the site of cancers registered from 1981 to 1999 initially used the ICD-9 system, but was recoded to the ICD-10 nomenclature by the Ministry of Health. The ICD-10 classification system was used from the calendar year 2000 onwards. Due to the effect of changes in classification for some subtypes of NHL [6], all types of NHL were combined.

Age-specific rates for men and women were calculated for each 5-year age group for the six 5-year time periods between 1981 and 2010 to create quin-quinquennial tables of rates. Age-standardised rates were calculated [11] using the WHO world standard population. Significance tests for trends in age-specific rates and age-adjusted trends over time used the Mantel-Haenszel extension test [12]. The associated statistical tests for linearity and nonlinearity were 2-sided and used the 5% level of significance.

Each rate in the quin-quinquennial tables of rates represents the experience of generations of several adjacent individual years of birth—labelled using the median year of birth [7]. A Poisson maximum likelihood APC model [13] derived from a previous weighted least squares method [14] was applied to the quin-quinquennial tables of NHL mortality and incidence rates, separately, for men and women aged 25 or more years. Registrations and mortality from NHL at younger ages were too infrequent for inclusion in the APC models fitted. When major time period or birth cohort (generation) effects are present and separable, and the weighting of the solution by the results of submodels is appropriate [14, 15], this APC model summarises the trends in the quin-quinquennial tables of rates. The model provides age effects that can be interpreted as average age-specific rates adjusted for time period and birth cohort effects, time period effects that estimate the relative risk (RR) for a time period relative to an average risk over the total time period studied, and birth cohort effects that estimate the RR for a birth cohort compared to an average birth cohort [13].

## 3. Results

**Figure 1:**
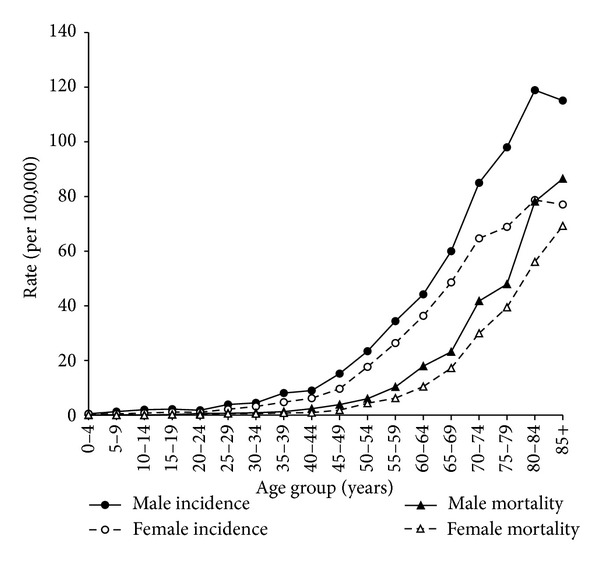
The male and female age-specific incidence and mortality rates of NHL for the 2001–2010 time period.

The age distributions of NHL incidence and mortality for the 2001–2010 time period are shown in Figure 1. The age-specific rates increased with age except for the incidence rates of the oldest age group for both males and females and were higher for males for each age group.

In 2006–2010, the average number of deaths attributed to NHL in New Zealand was 268 per year (152 men and 116 women), with age-standardised mortality rates per 100,000 of 4.5 for men and 3.0 for women. In 2006–2010, the age-standardised mortality rate was 13% higher in men (rate ratio = 1.13, 95% CI 0.95–1.34) and 18% higher in women (rate ratio = 1.18, 95% CI 1.00–1.40) compared to the 1981–1985 time period. In 2006–2010, the average number of registrations of NHL was 726 per year (400 men and 326 women), with age-standardised incidence rates per 100,000 of 13.9 for men and 9.7 for women. These incidence rates were 67% higher in men (rate ratio = 1.67, 95% CI 1.41–1.98) and 74% higher in women (rate ratio = 1.74, 95% CI 1.47–2.05) than for the 1981–1985 time period. Between the 1981–1985 and 2006–2010 time periods, for both sexes combined, the incidence of follicular NHL (ICD-10 rubric C82), diffuse NHL (C83), and peripheral and cutaneous T-cell NHL (C84) significantly increased by 120%, 63%, and 415%, respectively, while a more modest increase for other and unspecified NHL (C85) of 52% was observed (data not shown).

**Figure 2:**
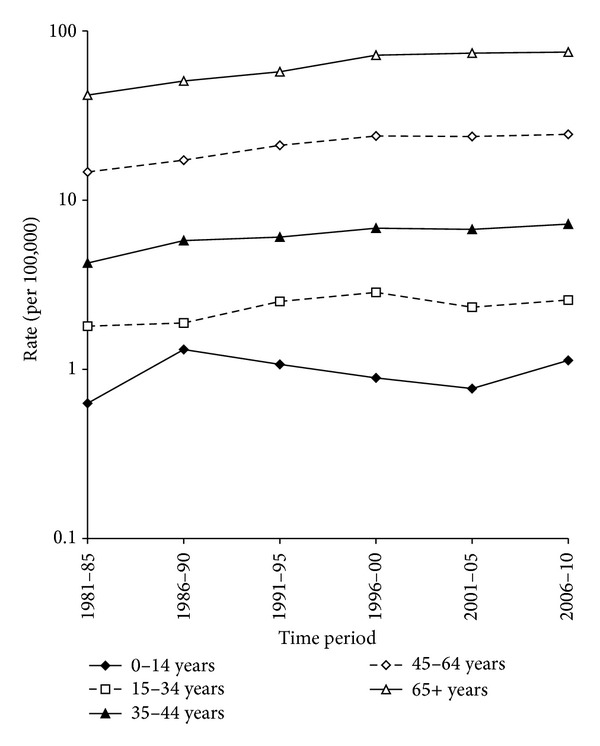
The trends in NHL incidence for broad age groups, both sexes combined.

The trends in incidence and mortality for males and females were similar, so the trends in NHL incidence for broad age groups, 0–14 years, 15–34 years, 35–44 years, 45–64 years, and 65 or more years of age were combined for both sexes (Figure 2). In children of 0–14 years of age, the incidence rate was the highest in the 1986–1990 time period, there was little overall increase in incidence (test for linear trend, *P* = 0.54), and there was a significant nonlinear trend over time (test for nonlinear trend, *P* = 0.01). In those 15–34 years of age, the incidence rate increased from 1.8 to 2.6 per 100,000 (test for linear trend, *P* = 0.001) but in a nonlinear manner (test for nonlinear trend, *P* = 0.04). For those 35–44 years of age, the incidence rate increased linearly from 4.3 to 7.2 per 100,000 (test for linear trend, *P* < 0.001). For those 45–64 and 65 or more years of age, the incidence rate increased nonlinearly from 14.7 to 24.5 and 41.8 to 75.0 per 100,000, respectively (test for nonlinear trend, *P* < 0.01 for both age groups).

**Figure 3:**
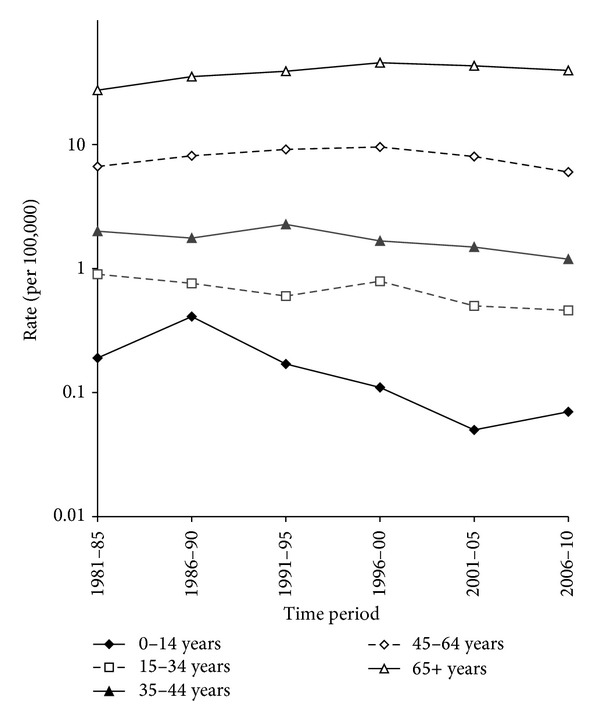
The trends in NHL mortality for broad age groups, both sexes combined.

For children of 0–14 years of age, the mortality rate of NHL was the greatest in the 1986–1990 time period and declined thereafter (Figure 3). For those 15–34 and 35–44 years of age, mortality from NHL decreased considerably between 1981–1985 and 2005–2009, from 0.9 to 0.5 and 2.0 to 1.2 per 100,000, respectively (*P* < 0.01 for both age groups) with little evidence of a nonlinear trend. The decreased mortality in these age groups was the greatest from 1996–2000 onwards. For those aged 45–64 years and 65 or more years, mortality from NHL increased between 1981–1985 and 1996–2000, from 6.7 to 9.6 and 27.4 to 45.6 per 100,000, respectively. The mortality rates for these age groups decreased significantly thereafter to 6.0 and 39.6 per 100,000, respectively, for the 2006–2010 time period. The trends in mortality rates were significantly nonlinear (test for nonlinear trend, *P* < 0.001 for both age groups).

In 2006–2010, the age-adjusted male to female incidence rate ratio was 1.44 (95% CI 1.35–1.53). However, for each age group, 5–9 to 20–24 years, the male to female incidence rate ratio was greater than two in the 2006–2010 time period, consistently greater than for earlier time periods (data not shown).

**Figure 4:**
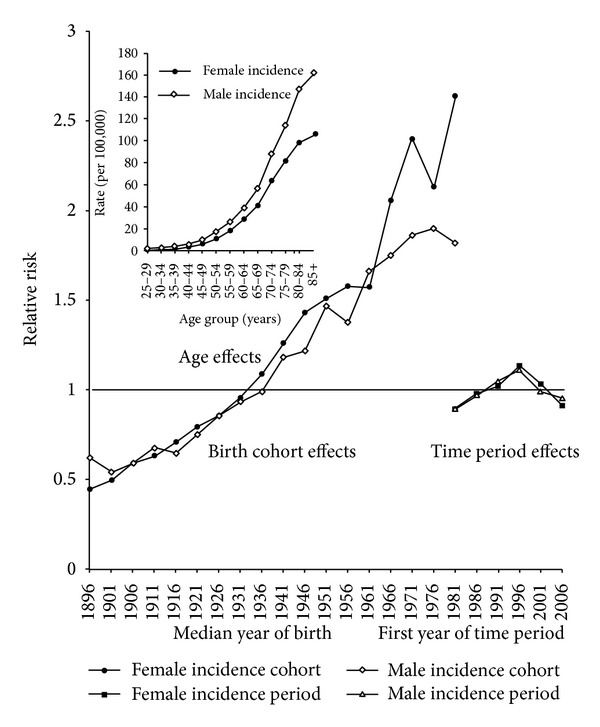
Age, time period, and birth cohort effects of trends in NHL incidence.

The age, time period, and birth cohort effects of NHL incidence for men and women, as estimated by the independent APC models, are plotted in Figure 4. The risk of a diagnosis of NHL in men and women, relative to an average birth cohort, increased considerably for men and women born from about 1901 to about 1971 with a possible brief slowing of the increase for generations born about 1956 for men and 1961 for women. For generations born from about 1971, the increase for successive generations was greater for women than for men. Fluctuations in risks for generations born after about 1971 are likely to be due to the low frequency of diagnoses of NHL in the age groups available. The dramatic increase in relative risk of NHL among generations born between about 1901 and those born about 1971 suggested, from the ratio of the relative risks, that recent generations have about five times the risk of NHL compared to generations born in the early 1900s.

**Figure 5:**
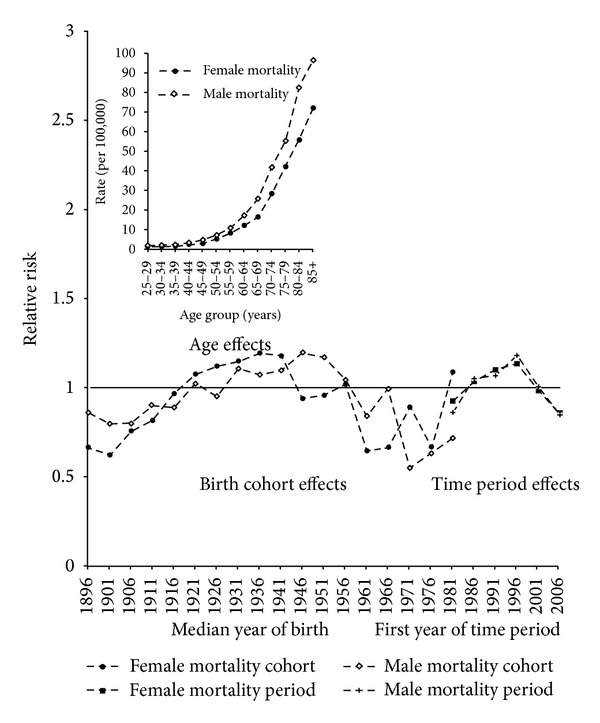
Age, time period, and birth cohort effects of trends in NHL mortality.

The results of the APC model for NHL mortality are plotted in Figure 5. The relative risk for mortality increased for successive generations of women born from about 1901 to 1936, decreased for women born about 1946, and fluctuated with an overall downward trend in relative risk for generations born thereafter. The changes in the estimates of the relative risk of NHL mortality for different generations of men fluctuated more than for women, increasing overall for men born between about 1901 and 1946 and generally decreasing for subsequent generations. The fluctuations in relative risks for the most recent generations of men and women, and to a lesser extent the earliest generation, are likely to be due to infrequent deaths in the most recent younger age groups and also in the oldest age group of the earliest time period. The time period effects for mortality for men and women increased between the 1981–1985 and 1996–2000 time periods and declined thereafter, a trend similar to that seen for incidence (Figure 4). The consistency of the changes in time period effects for incidence and mortality suggested that changes in the classification of NHL resulted in an increase in the incidence and mortality of NHL of between 24 and 37% in 1996–2000 compared to the 1981–1985 time period.

From the earliest generations shown, to those born about 1926, the increasing relative risk of NHL mortality and incidence compared to an average generation was similar for both men and women. However, while the relative incidence of NHL for successive generations continued to increase, the relative increase became less pronounced for mortality, and eventually declined. This has produced considerably higher incidence relative to mortality among generations born since about 1936 for women and about 1946 for men.

## 4. Discussion

As has occurred in many countries [16–19], the incidence of NHL for those 15 or more years of age in New Zealand has increased considerably, with significant increases in incidence for each of follicular NHL, diffuse NHL, and peripheral and cutaneous T-cell NHL and for other and unspecified NHL in New Zealand. We have found that the incidence of NHL in New Zealand has increased considerably for successive generations among women and men born since about 1936 and about 1946, respectively—a trend not seen for the mortality from NHL. Increases in the incidence of NHL for different generations have also been observed in other countries [16–18, 20].

The time period effects of the independent APC models of incidence and mortality for men and women suggested that changes in the classification of NHL may have produced a 24–37% alteration in NHL incidence and mortality since the 1981–1985 time period, much less than the 67% and 74% overall increase in incidence observed, respectively, for men and women. Changes in the classification of NHL over time have not been found to explain similar trends in other countries [17, 20, 21].

APC models can improve the assessment of trends in rates of NHL [8], particularly when changes in the classification of NHL or completeness of cancer registration have occurred. Effect estimates from APC models are dependent on an appropriate partial resolution to their inherent identification problem [14, 22], but nonlinear changes in the effects are uniquely determined [15] so that overall nonlinearity in birth cohort effects and time period effects, as we have observed for NHL incidence and mortality, are unlikely to be due to APC model selection.

The pronounced generation effects indicate that exposure to a major causal environmental factor has occurred in utero, during childhood, or youth before 25 years of age. Alternatively, the prevalence of a protective agent may have declined. Helicobacter pylori infection is associated with rare gastric MALT lymphoma, which can regress with eradication of the infection, indicating that an infectious agent may be an important cause of some forms of NHL [2]. If contact with some infectious agent early in life was protective for NHL [2], older age of infection or lower infection rates over successive decades may have lowered that protection.

An increased risk of NHL has been found to be associated with many immunological disorders, including autoimmune immunodeficiency syndrome (AIDS). As in other countries, most of the generations for whom an increased risk of NHL was observed were not young when AIDS became apparent in New Zealand, so the contribution of AIDS to the increasing incidence of NHL is likely to be negligible. While the prevalence of asthma is considered to have increased in New Zealand, the contribution of asthma and other allergic conditions to the trends in the incidence of NHL is unknown [2].

The lack of an overall increase of NHL in childhood suggests that either different factors at a young age or in utero are important for the development of NHL in youth compared to adulthood or the increasing risk for successive generations of adults will become ameliorated in the future as these children age. The divergent generation effects for incidence compared to mortality suggest that much of the increase in NHL for recent generations may be due to the least fatal forms of the disease or NHL has been diagnosed when it is more amenable to cure or improved treatment has been introduced for successively younger patients over time.

The trends observed suggest that the epidemic of adult NHL will continue to occur in New Zealand over at least the next three decades, as those generations identified with increased lifetime risk get older. Moreover, the greater increase in relative risk for generations of women born from about 1966 to 1981 compared to men suggested that exposure to a causative agent increased more in girls than boys, or conversely exposure to a protective agent increased more in boys than girls for these generations. However, the increased male to female incidence rate ratio for those 5 to 24 years of age in the most recent time period suggests that the difference in prevalence between boys and girls of such a factor may have decreased again for generations born since about 1986.

Some slowing of the epidemic may be possible, as a similar epidemic of NHL in Scandinavia is thought to be resolving, as recent generations have not continued the increased lifetime risk of earlier generations [18], but the causes of the epidemic or its amelioration remain unknown [2].

It has been suggested that the divergence between trends in incidence and mortality from NHL in the United Kingdom, especially at younger ages, was due to the introduction of effective chemotherapy and radiotherapy more aggressively and successfully at younger ages [20]. New therapies introduced at successively younger 5-year age groups over successive 5-year time periods over 10 to 15 years could explain the different experience of NHL among generations observed in New Zealand and contribute to the divergent trends observed in incidence and mortality for successive generations. The increased incidence has occurred for most subtypes of NHL, so it is possible that earlier diagnosis, sufficient to result in the prevention of death from NHL, occurring for successive generations may also explain some of the trends observed. Further assessment of the impact of changes in treatment and presentation of NHL in New Zealand is warranted.

The increased incidence of NHL induced by chemotherapeutic drugs and immunosuppression therapy following bone marrow or other organ transplantation is likely to contribute little to the increased incidence of NHL. This is supported by several studies that have found that the incidence of NHL among patients with other cancers has not increased more than the overall incidence of NHL [2, 18, 21]. It is possible that technological advances may have resulted in more biopsies or lymph node resections when investigating illness, resulting in more diagnoses of occult NHL. However, this is unlikely to explain the observed increase in incidence among those less than 45 years of age.

The many types of lymphoma appear to have different risk factors. For example, late birth order and high body mass index have been shown to be associated with increased risk of diffuse large B-cell lymphoma, whereas autoimmune conditions have been associated with an increased risk of marginal zone lymphoma [23]. Analyses by the Interlymph and EpiLymph consortia, combining the results of several studies, have suggested a reduced risk of specific B-cell subtypes from increased recreational sun exposure [24, 25]. Further exploration of the potential role of UV radiation in New Zealand may be warranted, despite the low relative risks associated with increased sun exposure. Where trends in subtypes of NHL have been examined, the increase in the incidence of NHL has been found to be the greatest for follicular, extranodal B-cell and nodal T-cell lymphoma [17]. Detailed examination of trends by NHL subtype in New Zealand is needed.

Many risk factors have been suggested for NHL and its subtypes [2] but, to explain the observed epidemic of NHL in New Zealand and elsewhere, the relative risks of NHL from putative exposures would need to be much larger than those found for most risk factors. In addition, the prevalence of exposure would need to have changed considerably for successive generations. For example, the use of hair dyes has been suggested as a cause of NHL, but the association has been inconsistent [2] and is unlikely to explain the male predominance of the disease or the trends observed. In addition, despite New Zealand having an extensive agricultural and horticultural workforce historically involving significant use of various herbicides and pesticides, these exposures have not been consistently found to increase the risk of NHL [2].

## 5. Summary

The epidemic of NHL occurring in New Zealand is not explained by our current knowledge of the possible causes of NHL or any of its subtypes and new testable hypotheses of causation are needed to explain the trends observed. Differences in incidence patterns by histologic subtype would be suggestive of etiologic heterogeneity among lymphoid neoplasms and support the pursuit of epidemiologic analysis by subtype. Further research to understand and control this epidemic of NHL in New Zealand is required.
